# Microbiota and inflammatory factor profiles in different stages of bacterial vaginosis formation and recurrence

**DOI:** 10.1099/jmm.0.002183

**Published:** 2026-08-07

**Authors:** Qing Zhu, Yutong Li, Hu Luo

**Affiliations:** 1 Department of Clinical Laboratory Diagnostics, Beijing Friendship Hospital, Capital Medical University, Beijing 100050, PR China

**Keywords:** 16S-rRNA sequencing, bacterial vaginosis, recurrence, vaginal microbiota

## Abstract

**Introduction.** Bacterial vaginosis (BV) is a common gynaecological disorder characterized by an imbalance in the vaginal microbiota, leading to increased risk of infection and recurrence.

**Hypothesis/Gap statement.** Although the role of microbiota dysbiosis in BV is established, the dynamic changes in microbial communities and inflammatory factors during BV formation and recurrence, as well as reliable biomarkers for predicting recurrence, remain inadequately explored.

**Aim.** This study aimed to systematically analyse the changes in vaginal microbiota and inflammatory factors during different stages of BV formation and recurrence, and to identify potential biomarkers for predicting BV recurrence.

**Methodology.** Vaginal swab samples were collected from 135 women, including 55 BV patients, 50 intermediate BV (IBV) patients and 30 healthy controls. Twenty-six recurrent BV (Re-BV) samples were obtained within 1 year after treatment. 16S rRNA gene sequencing was performed to profile the vaginal microbiota, and ELISA was used to measure IL-6, IL-10 and IL-1β levels. Statistical analyses included diversity metrics, correlation analysis and predictive modelling using LASSO regression and receiver operating characteristic curves.

**Results.** Microbial richness was significantly higher in BV, Re-BV and IBV groups compared to controls. *Lactobacillus* abundance decreased progressively from IBV to BV, while *Gardnerella*, *Prevotella*, *Sneathia*, *Fannyhessea* and *Dialister* increased. These genera were positively correlated with vaginal pH, human papillomavirus (HPV) status and enzymatic activity. IL-6, IL-10 and IL-1β levels were elevated in BV, IBV and Re-BV groups. IL-10 and IL-1β were further increased in recurrent cases. A combined predictive model integrating microbial and cytokine markers achieved an area under the curve of 0.928 for BV recurrence.

**Conclusion.** Distinct shifts in vaginal microbiota composition and inflammatory factor levels occur during BV formation and recurrence. The integration of microbial and cytokine profiles offers a robust approach for predicting BV recurrence, with important implications for clinical management and prevention strategies.

## Data Summary

Datasets for this current study can be found under BioProject number PRJNA1161362. Individual sample accession numbers can be found in Table S19, available in the Supplementary Material.

## Introduction

Vaginosis is a disease with a high prevalence in gynaecological outpatient clinics, accounting for more than 30% of gynaecological diseases [1–3]. Bacterial, fungal and viral infections are the main causes of vaginosis [4, 5]. The incidence rate of bacterial vaginosis (BV) is significantly higher than that of fungal and viral infections [6]. The main components of female vaginal microbiota include Gram-positive anaerobic bacteria, such as *lactobacilli*, *streptococci* and *Escherichia coli*, as well as aerobic bacteria [7–9]. The imbalance of microbiota is a typical feature of BV and the persistent colonization of *Gardnerella* is one of the common hallmarks of BV, but changes in other anaerobic bacteria remain unclear. Therefore, exploring the changes of vaginal microbiota is of great significance.

Intermediate BV (IBV) is a transitional stage in BV formation. At present, it is common in clinical practice to distinguish BV and IBV based on the Nugent score, but this judgement criterion has significant subjective factors. At present, there is a lack of relevant research on the changes in the microbiota during the IBV stage. In addition, the recurrence of BV is a clinical treatment challenge for this disease. Antibiotics are the first-line treatment for BV in clinical practice, and metronidazole is the representative drug [10–12]. However, the use of antibiotics can also lead to changes in vaginal microbiota, leading to disease recurrence [13–15]. Therefore, exploring the changes of the microbiota from the onset of vaginosis to the recurrence process is beneficial for us to better treat and prevent the disease.

In addition to the impact of changes in the microbiota on vaginosis, the local vaginal immunity also plays an important role in limiting and eliminating microbiological infections [16]. Various cytokines, complements and immunoglobulins in the vaginal mucosa form the first line of defence against the invading pathogens [17]. Studies have demonstrated an impairment of the mucosal immune system in women with BV as evidenced by degradation of IgA and IgM in the vaginal washes [18]. Different cytokines and chemokines are influenced by individual bacterial taxa, which may differ between women with BV [19]. *Lactobacillus* promotes the significant increase in IP-10 and reduces the level of IL-12, IL-8 and IL-1β, which could possibly have played an important role in defending against pathogenic bacteria [20]. To date, multiple studies have shown that pro-inflammatory molecules, including IL-1, IL-6 and IL-8, are closely related to the formation of BV, but there is limited research on the changes of inflammatory factors in the progression of BV.

In this study, we systematically analysed the changes in microbiota and inflammatory factors during the stages of BV formation and recurrence and searched for biomarkers to predict BV recurrence.

## Methods

### Patients and sample collection

Vaginal discharge samples were collected from 135 patients at the Gynaecology Outpatient Department of Beijing Friendship Hospital, Capital Medical University between January 2023 and June 2023, including 55 BV patients, 50 IBV patients and 30 healthy volunteers. The majority of participants were of Han Chinese ethnicity, reflecting the local demographic composition. The swabs of 26 patients with recurrent BV (Re-BV) within 1 year after treatment were collected again. The inclusion criteria of the patients were as follows: (1) patients aged 18–45 years; (2) all women had a sexual history and were not pregnant; (3) patients had no history of antibiotic use in the past week, and have not taken oestrogen or progestogen in the past 3 months; (4) there has been no sexual activity, no history of vaginal flushing or medication, and menstruation has been clean for at least 3 days; (5) following the collection of the biological samples, all the BV patients were prescribed 400 mg metronidazole tablets, with one tablet to be taken twice daily, patient adherence was self-reported. The normal control (NC) group consisted of healthy volunteers with no clinical symptoms of vaginal infections, a Nugent score of 0–3, normal vaginal pH (≤4.5) and no history of antibiotic or hormonal therapy within the past 3 months, as confirmed by gynaecological examination and microbiological assessment. The exclusion criteria were as follows: (1) patients who exhibit fungal spores or hyphae as shown by Gram staining on secretion smear; (2) DNA extraction failed or quality control failed due to drying of clinical swab samples. Clinical diagnosis of BV (7≤Nugent score ≤10) and IBV (4≤Nugent score ≤6) was performed through Nugent scoring. All vaginal smear slides for Nugent scoring were independently evaluated by two experienced pathologists who were blinded to the participants' clinical information. Discrepancies with a score difference ≥2 were resolved by re-evaluation by a third senior pathologist to reach a consensus, ensuring the reliability of diagnostic classification. This study was reviewed and approved by the Ethics Committee of Beijing Friendship Medical College Affiliated Capital Medical University (Approval No. 2023-P2-054-01).

### DNA extraction

The vaginal swab was first soaked in physiological saline, and the secretion was eluted into the liquid. DNA was extracted with the Qiagen’s DNA Mini Kit (Qiagen, Hilden, Germany) according to the manufacturer’s instructions. The DNA concentration of the samples was measured with the Qubit dsDNA HS Assay Kit and Qubit 4.0 Fluorometer (Invitrogen, Thermo Fisher Scientific, Oregon, USA).

### ELISA

ELISA was performed to determine the levels of IL-10, IL-1β and IL-6 in vaginal secretions. Briefly, vaginal discharge was suspended in physiological saline and the supernatants were harvested via centrifugation at 5,000 r.p.m. for 5 min. The expression levels of IL-10, IL-1β and IL-6 were detected using ELISA kits (cat no. PI528, PI305, PI330, Beyotime Institute of Biotechnology) according to the manufacturer’s protocols.

### Amplicon sequencing

The 338F: 5′-ACTCCTACGGGAGGCAGCA-3′ and 806R: 5′-GGACTACHVGGGTWTCTAAT-3′ universal primer set was used to amplify the V3-V4 region of the 16S rRNA gene from the genomic DNA extracted from each sample. Both the forward and reverse 16S primers were tailed with sample-specific Illumina index sequences to allow for deep sequencing. The PCR was performed in a total reaction volume of 10 µl DNA template (25 ng), 0.3 µl forward primers (10 µM), 0.3 µl reverse primers (10 µM), 5 µl KOD FX Neo Buffer, 2 µl dNTP (2 mM), 0.2 µl KOD FX Neo and ddH2O to a final volume of 10 µl. Amplification started with initial denaturation at 95 °C for 5 min, followed by 25 cycles of denaturation at 95 °C for 30 s, annealing at 50 °C for 30 s and extension at 72 °C for 40 s and a final step at 72 °C for 7 min. The total of PCR amplicons were purified with Agencourt AMPure XP Beads (Beckman Coulter, Indianapolis, IN, USA) and quantified using the Qubit dsDNA HS Assay Kit and Qubit 4.0 Fluorometer (Invitrogen, Thermo Fisher Scientific, Oregon, USA). After the individual quantification step, amplicons were pooled in equal amounts. The constructed library was sequenced using Illumina NovaSeq 6000 (Illumina, Santiago, CA, USA).

To minimize potential contamination, negative controls were included throughout the experimental workflow: extraction blanks (sterile saline processed alongside samples during DNA extraction) and PCR blanks (no template DNA added to PCR reactions). These controls were processed alongside samples throughout the entire workflow (DNA extraction, PCR amplification and sequencing).

### Operational taxonomic unit cluster and species annotation

Raw data were primarily filtered by Trimmomatic. Primer sequences were identified and removed using Cutadapt. PE reads obtained from previous steps were assembled by USEARCH and followed by chimaera removal using UCHIME. The high-quality reads generated from above steps were used in the following analysis. Sequences with similarity ≥97% were clustered into the same operational taxonomic unit (OTU) by USEARCH, and OTUs with relative abundance <0.005% were filtered. Taxonomy annotation of the OTUs was performed based on the Naive Bayes classifier in QIIME2 using the silva database. According to the optimal parameter recommendations for 16S rRNA gene short amplicons [21], a default confidence threshold of 0.7 (70%) was applied for the classification to maintain an optimal balance between precision and recall. Furthermore, to ensure data reliability, only OTUs that were successfully annotated at the genus level were retained for subsequent differential screening and predictive modelling. Following this, we employed linear discriminant analysis (LDA) effect size (LEfSe) to test the significant taxonomic difference among groups. A logarithmic LDA score of 4.0 was set as the threshold for discriminative features. To explore the dissimilarities of the microbiome among different factors, a redundancy analysis was performed in R using the package ‘vegan’.

### Microbiota diversity analysis

Alpha diversity was calculated and displayed using QIIME2 and R software, respectively, which were applied to analyse the complexity of species diversity for each sample through Chao1, Shannon index and Faith’s Phylogenetic Diversity. Beta diversity was evaluated to assess the structural variation of microbial communities across different groups. Principal coordinate analysis (PCoA) was performed based on Bray-Curtis, Unweighted UniFrac and Weighted UniFrac distance metrics. To determine the statistical significance of structural differences among groups, permutational multivariate analysis of variance (PERMANOVA) with 999 permutations was conducted using the ‘vegan’ package in R. Furthermore, pairwise PERMANOVA with false discovery rate correction was performed to identify specific inter-group differences.

### Statistical analysis

Descriptive statistics were performed to summarize the data. Quantitative data were shown as median, and categorical data were shown as n (%). The Kolmogorov-Smirnov test was used to assess the normality of the data distribution. For comparisons between groups, one-way ANOVA was used for quantitative data, and Fisher’s exact test was used for categorical data. The Wilcoxon rank-sum test was used to evaluate the statistical differences in alpha diversity, including the Chao1 index and Shannon index. An unpaired t-test was performed to compare the concentrations of inflammatory factors between groups. The Spearman rank correlation coefficient was used to analyse the correlations between microbial abundance and inflammatory factor levels. The receiver operating characteristic curve (ROC) analysis was performed to assess the predictive accuracy of the inflammatory factors. Statistical significance was indicated as follows: *P<0.05, **P<0.01, ***P<0.001, ****P<0.0001.

### Construction and validation of a recurrence prediction model

Microbiome data were first transformed into relative abundances and subsequently standardized. LASSO regression with lambda.min was applied for variable selection, and the selected microbial features were used to construct predictive models. ROC curves were plotted and the area under the curve (AUC) was calculated to evaluate model performance. For cytokine data, variables were standardized and directly entered into multivariable regression models, followed by ROC analysis and AUC calculation. Finally, a combined multivariable regression model was constructed using both the LASSO-selected microbial features and selected cytokine variables. To assess the generalizability of the model and mitigate the potential risk of overfitting due to the limited sample size, leave-one-out cross-validation (LOOCV) was performed on the current dataset. A calibration curve was plotted to assess the agreement between the model’s predicted probabilities and the actual observed outcomes. Furthermore, the clinical utility of the predictive model was evaluated using decision curve analysis (DCA) to estimate the net benefit at different threshold probabilities. To evaluate the specific predictive contributions of the selected features while mitigating the risk of mathematical instability caused by data sparsity (e.g. complete separation) in the recurrent cohort, Ridge regularized regression (L2 penalty) was applied. The extracted coefficient weights were normalized to Relative Feature Importance (0–100%) to provide a robust ranking of each microbe and cytokine’s contribution to the recurrence prediction.

## Results

### Vaginal swab samples and patient characteristics

A total of 135 patients were included in the study and divided into three groups. There was no difference in age between the three groups. The average pH value of the BV group was 4.88, which is very close to the IBV group and higher than the NC group. 21.8% of patients in the BV group had human papillomavirus (HPV) infection and 10% of patients in the IBV group were HPV positive, indicating that HPV infection may be closely related to vaginal microbial imbalance. In addition, the positivity of neuraminidase, acetylglucosaminidase, hydrogen peroxide and leucocyte esterase is a significant characteristic of patients with BV and IBV. 47.3% of the 55 BV patients experienced recurrence within 1 year after treatment with metronidazole, and these patients were classified as the Re-BV group. The clinical information and vaginal microenvironment characteristics of all patients are presented in Table 1.

**Table 1.** General characteristics of participants

Characteristics	BV (*n*=55)	IBV (*n*=50)	NC (*n*=30)	*P-*value
Age (years)	41.29	40.48	39.37	0.7336
pH	4.88	4.89	4.46	<0.0001
HPV subtype (n/%)				0.0428
HPV-16	2 (3.64)	1 (2.0)	0	
HPV-18	1 (1.82)	1 (2.0)	0	
HPV-other types	9 (16.36)	3 (6.0)	1 (3.33)	
Hydrogen peroxide + (n/%)	49 (89.09)	49 (98.0)	21 (70.0)	0.0010
Leucocyte esterase + (n/%)	16 (29.09)	11 (22.0)	1 (3.33)	0.0099
Neuraminidase + (n/%)	38 (69.09)	10 (20.0)	0	<0.0001
β-glucuronidase + (n/%)	0	0	0	ns
Acetylglucosaminidase + (n/%)	27 (49.09)	18 (36.0)	2 (6.67)	0.0002

Quantitative data were shown as median, and categorical data were shown as n (%). One-way ANOVA analysis was used for the statistical analysis of quantitative data; Fisher’s exact test was used for the statistical analysis of categorical data.

### Vaginal microbiome diversity between the BV, Re-BV, IBV and NC groups

**Fig. 1.**
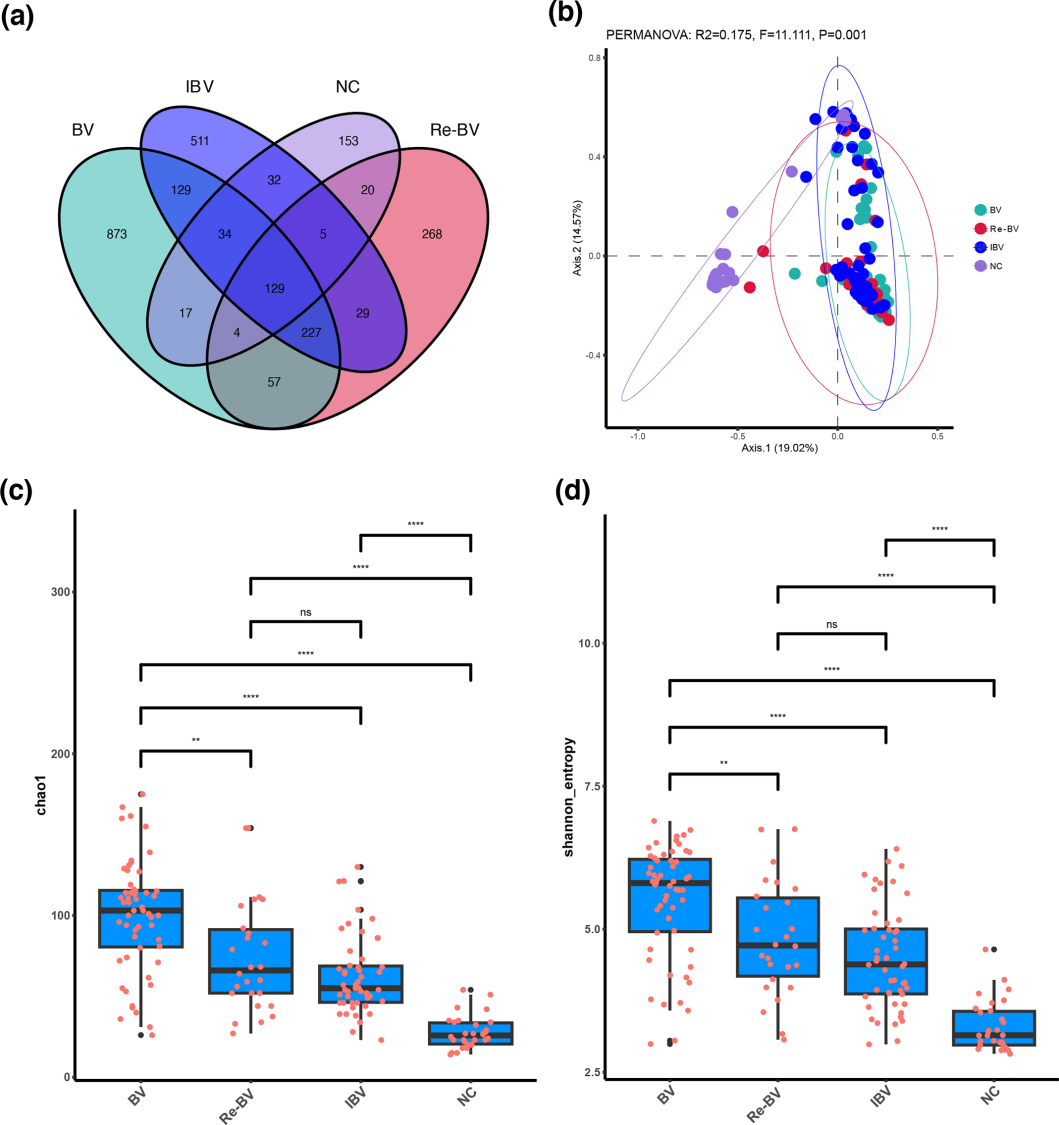
Diversity analysis of vaginal microbiota among BV, Re-BV, IBV and NC groups. (**a**) ASVs Venn diagram of the four groups. (**b**) PCoA analysis was based on the Bray-Curtis distance among the four groups. (**c**) Shannon index among BV, Re-BV, IBV and NC groups at the OTU level. (**d**) Simpson index among BV, Re-BV, IBV and NC groups at the OTU level.

In order to compare the differences in vaginal microbial diversity of each group, we identified the characteristic sequences of each group. The Venn diagram revealed that there were 129 shared amplicon sequence variants (ASVs) within the samples of the four groups. BV, Re-BV, IBV and NC group had 873, 268, 511 and 153 unique ASVs, respectively, indicating that the microbiota community of each group was varied (Fig. 1a). The PCoA of Bray-Curtis distances was used to compare beta diversity among the four groups. The NC group was significantly separated from BV, Re-BV and IBV groups (PERMANOVA, R² = 0.230–0.266, adjusted *P*<0.01; Fig. 1b and Table S1). In contrast, BV and Re-BV groups were not clearly separated (R² = 0.023, adjusted *P*<0.05) and there was no significant difference between Re-BV and IBV groups. To ensure the robustness of our findings, we further validated the vaginal microbial community structural variation using phylogenetic-based Weighted and Unweighted UniFrac distances. The overall structural differences among groups were highly consistent with the Bray-Curtis analysis (all *P*<0.01; Fig. S2a and b). Furthermore, pairwise PERMANOVA confirmed that the phylogenetic composition underwent significant shifts across nearly all stages of BV formation and recurrence (Tables S2 and S3). These consistencies demonstrate the robustness of the results, while the minor differences between the algorithms reflect the characteristic evolution of bacterial abundance across various pathological stages. In addition, multiple parameters are used to evaluate the alpha diversity. The Chao1 index and Shannon index both indicated that the microbial richness of the BV, Re-BV and IBV groups was greater than that of the NC group, with statistical significance (Wilcoxon rank-sum test, U values for comparisons of Chao1 index: BV vs. NC: 1612.5, Re-BV vs. NC: 741.5, IBV vs. NC: 1420, *P*<0.0001; U values for comparisons of Shannon index: BV vs. NC: 1583, Re-BV vs. NC: 727, IBV vs. NC: 1345, *P*<0.0001; Fig. 1c–d).

### Microbial composition in BV, Re-BV, IBV and NC groups and associations with clinical parameters

**Fig. 2.**
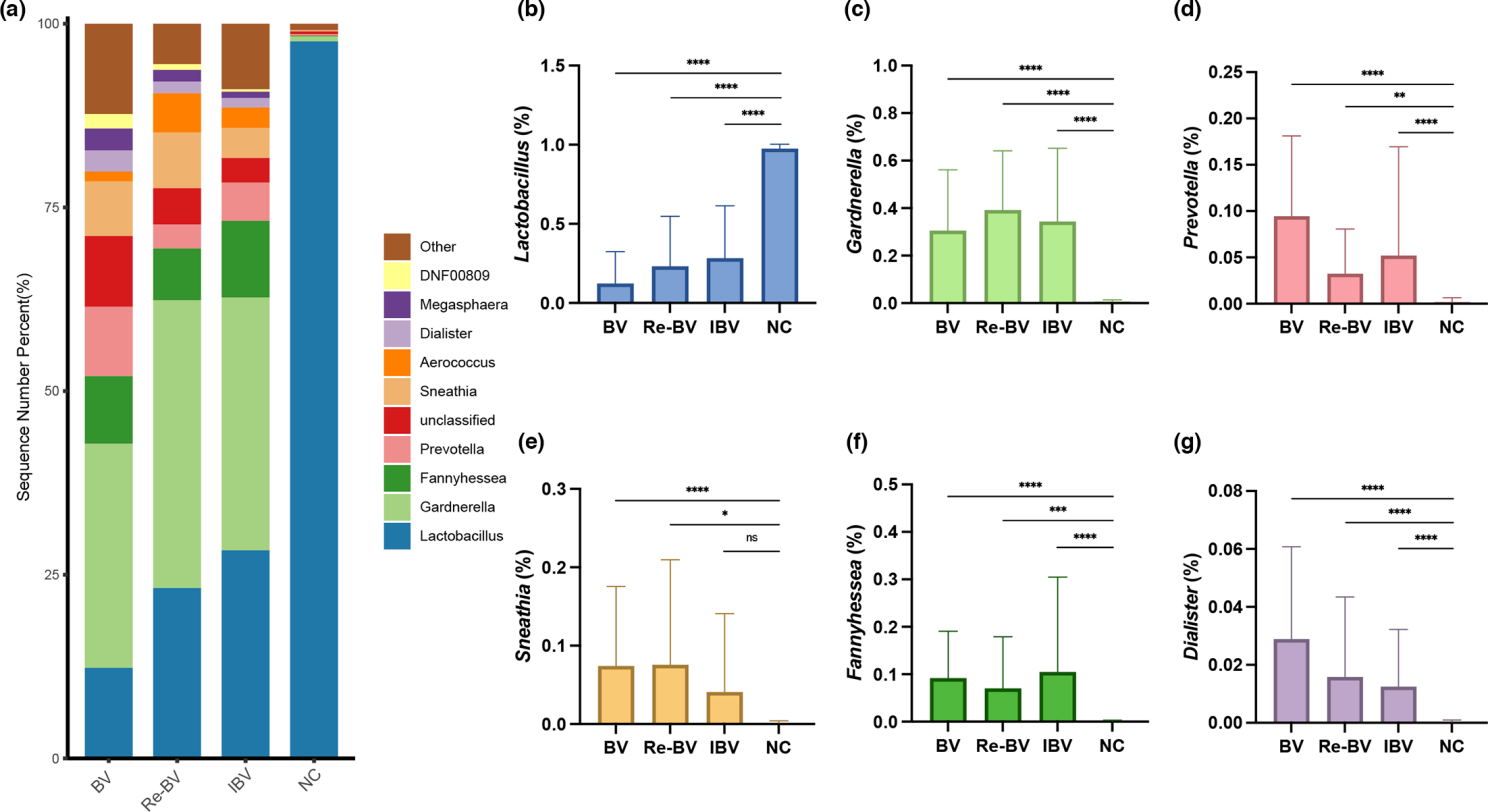
Microbial community structures and differences among BV, Re-BV, IBV and NC groups. (**a**) The microbial community bar plot of the four groups at the genus level. (**b-g**) The percentage changes of the microbial genera *Lactobacillus* (**b**), *Gardnerella* (**c**), *Prevotella* (**d**), *Sneathia* (**e**), *Fannyhessea* (**f**) and *Dialister* (**g**) in BV, Re-BV, IBV and NC groups.

*Lactobacillus* was the dominant genus in the control group, and the proportion of *Lactobacillus* in IBV, Re-BV and BV gradually decreased (Fig. 2a and b). The microbial profiling across the BV, Re-BV, IBV and NC groups revealed distinct patterns of distribution for the major bacterial taxa. *Gardnerella*, *Prevotella*, *Sneathia*, *Fannyhessea* and *Dialister* exhibited varying degrees of abundance. In the BV, IBV and Re-BV groups, there was a consistent increase in the relative percentages of these genera, while in the NC group, their relative abundance was particularly low (Fig. 2c–g).

To further clarify the associations between vaginal microbiota and key clinical parameters, we conducted correlation and group comparison analyses. Spearman correlation revealed that *Lactobacillus* abundance was significantly negatively correlated with vaginal pH (rho=−0.39, *P*<0.01), while *Gardnerella*, *Prevotella*, *Sneathia*, *Fannyhessea* and *Dialister* showed significant positive correlations with pH (all *P*<0.01; Table S4). No significant associations were found between genus abundance and age (Table S5).

Comparisons between HPV-negative and HPV-positive groups showed higher *Lactobacillus* abundance in HPV-negative samples (median 15.12% vs. 1.30%, *P*<0.01), while *Prevotella*, *Fannyhessea* and *Dialister* were more abundant in HPV-positive samples (*P*<0.05). *Gardnerella* and *Sneathia* did not differ significantly by HPV status (Table S6).

*Gardnerella* was significantly enriched in the hydrogen peroxide-positive group (median 24.39% vs. 4.13%, *P*<0.01), with no significant differences for other genera (Table S7). *Prevotella* and *Dialister* were more abundant in the leucocyte esterase-positive group (*P*<0.01), while other genera showed no significant differences (Table S8).

In the neuraminidase-positive group, *Gardnerella*, *Prevotella*, *Sneathia*, *Fannyhessea* and *Dialister* were significantly increased (*P*<0.05), and *Lactobacillus* was markedly decreased (median 2.87% vs. 41.70%, *P*<0.01; Table S9). Similarly, *Prevotella*, *Sneathia*, *Fannyhessea* and *Dialister* were enriched in the acetylglucosaminidase-positive group (*P*<0.01), with a significant reduction in *Lactobacillus* (median 3.04% vs. 35.96%, *P*<0.01). *Gardnerella* abundance was not significantly different by acetylglucosaminidase status (Table S10).

Overall, these results indicate that vaginal microbiota composition is closely linked to pH, HPV infection and specific enzymatic activities. *Lactobacillus* is consistently depleted in conditions associated with higher pH, HPV positivity and increased enzyme activity, while several anaerobic genera are enriched.

**Fig. 3.**
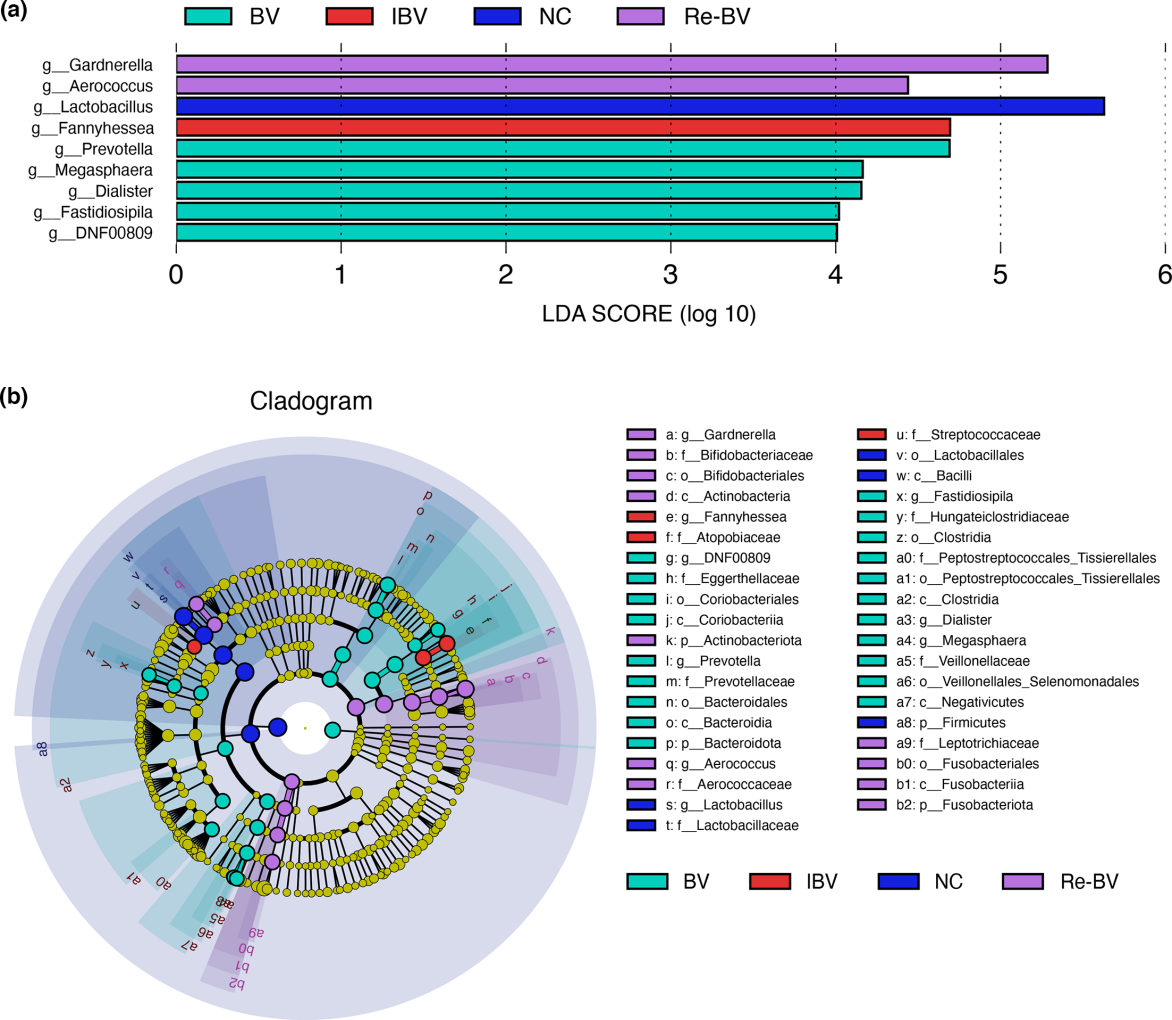
LEfSe analysis of differentially abundant genera among groups. (**a**) LDA bar plot showing significantly different genera and their LDA scores (threshold=4) in BV, Re-BV, IBV and NC groups. (**b**) Cladogram illustrating the phylogenetic relationships and distribution of dominant genera in each group.

To identify significantly different bacterial genera among groups, LEfSe analysis was performed with an LDA threshold of 4. A total of nine genera exhibited significant differences among the BV, Re-BV, IBV and NC groups. The BV group showed higher abundances of *Prevotella*, *Megasphaera*, *Dialister* and *Fastidiosipila. Gardnerella* and *Aerococcus* were significantly enriched in the Re-BV group, *Fannyhessea* in the IBV group and *Lactobacillus* dominated the NC group (Fig. 3a). The cladogram (Fig. 3b) further illustrates the phylogenetic relationships and distribution of these differential genera, highlighting distinct vaginal microbial community structures among groups.

### Differences in inflammatory factors among BV, Re-BV, IBV and NC groups

**Fig. 4.**
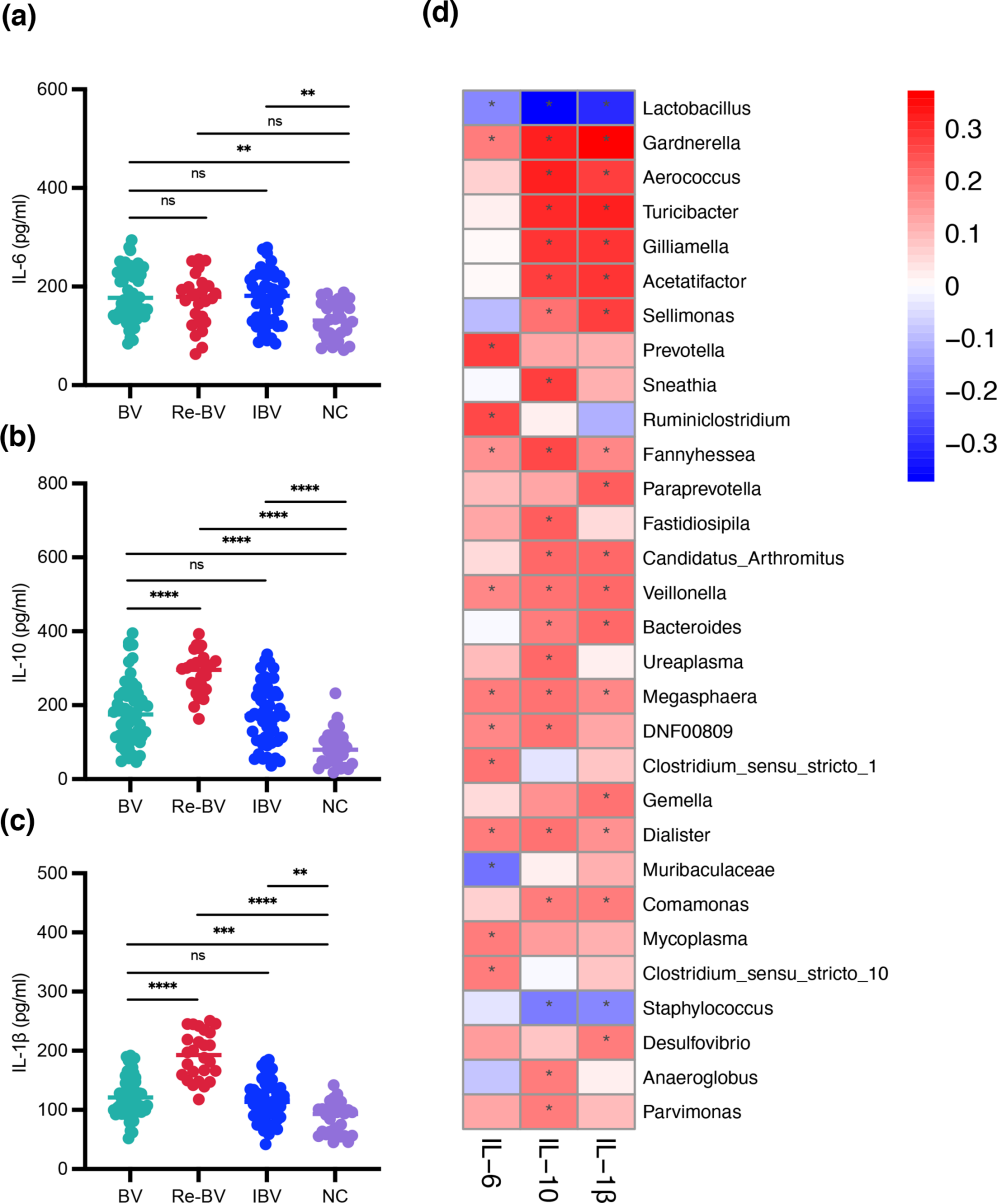
IL-6, IL-10 and IL-1β levels across different groups and correlation with microbial abundance. (**a**) The IL-6 concentration among BV, Re-BV, IBV and NC groups. (**b**) The IL-1β concentration among BV, Re-BV, IBV and NC groups. (**c**) The IL-10 concentration among BV, Re-BV, IBV and NC groups. (**d**) Heatmap of genus-level correlation between microbial abundance and the levels of IL-6, IL-10 and IL-1β.

Inflammatory factors play an important role in the formation of vaginosis. We detected changes of inflammatory factors in vaginal secretions of patients in different groups. Multiple linear regression analyses revealed that NC groups had significantly lower levels of IL-6 (mean difference=50.74, *P*=0.0005; Tables S11–S12), IL-10 (mean difference=104.8, *P*<0.0001; Tables S13–S14) and IL-1β (mean difference=37.66, *P*=0.0001; Tables S15–S16) compared to BV groups. Re-BV groups showed significantly higher levels of IL-10 (mean difference=100.5, *P*<0.0001; Table S14) and IL-1β (mean difference=69.46, *P*<0.0001; Table S16) than BV groups. Significant differences were also observed between IBV and NC groups for IL-6 (mean difference=41.04, *P*=0.0088; Table S12) and IL-1β (mean difference=29.99, *P*=0.0042; Table S16). No significant differences were found between BV and IBV groups for any cytokine (all *P*>0.05; Fig. 4a–c). Age, vaginal pH and HPV infection status did not significantly affect cytokine levels. To further elucidate these relationships, we used a heatmap to depict the correlation between microbial abundance and the levels of IL-6, IL-10 and IL-1β. The abundance of *Lactobacillus* was significantly negatively correlated with the concentration of IL-6, IL-10 and IL-1β (*P*<0.05). Conversely, the abundance of *Gardnerella*, *Fannyhessea* and *Dialister* were significantly positively correlated with the concentration of IL-6, IL-10 and IL-1β (*P*<0.05). The abundance of most microbial genera showed a strong correlation with the concentrations of the immunological markers IL-10 and IL-1β (Fig. 4d).

### Predictive modelling of BV recurrence based on microbiome and cytokine profiles

A total of 55 individuals diagnosed with BV, among whom 26 experienced recurrence, were included in this analysis. To identify potential microbial predictors of recurrence, LASSO regression was applied to the microbiome data, resulting in the selection of five genera: *Coriobacteriales* bacterium *DNF00809*, *Campylobacter*, *Howardella*, *Finegoldia* and *Proteus* (Fig. S1a and b; Table S17).

**Fig. 5.**
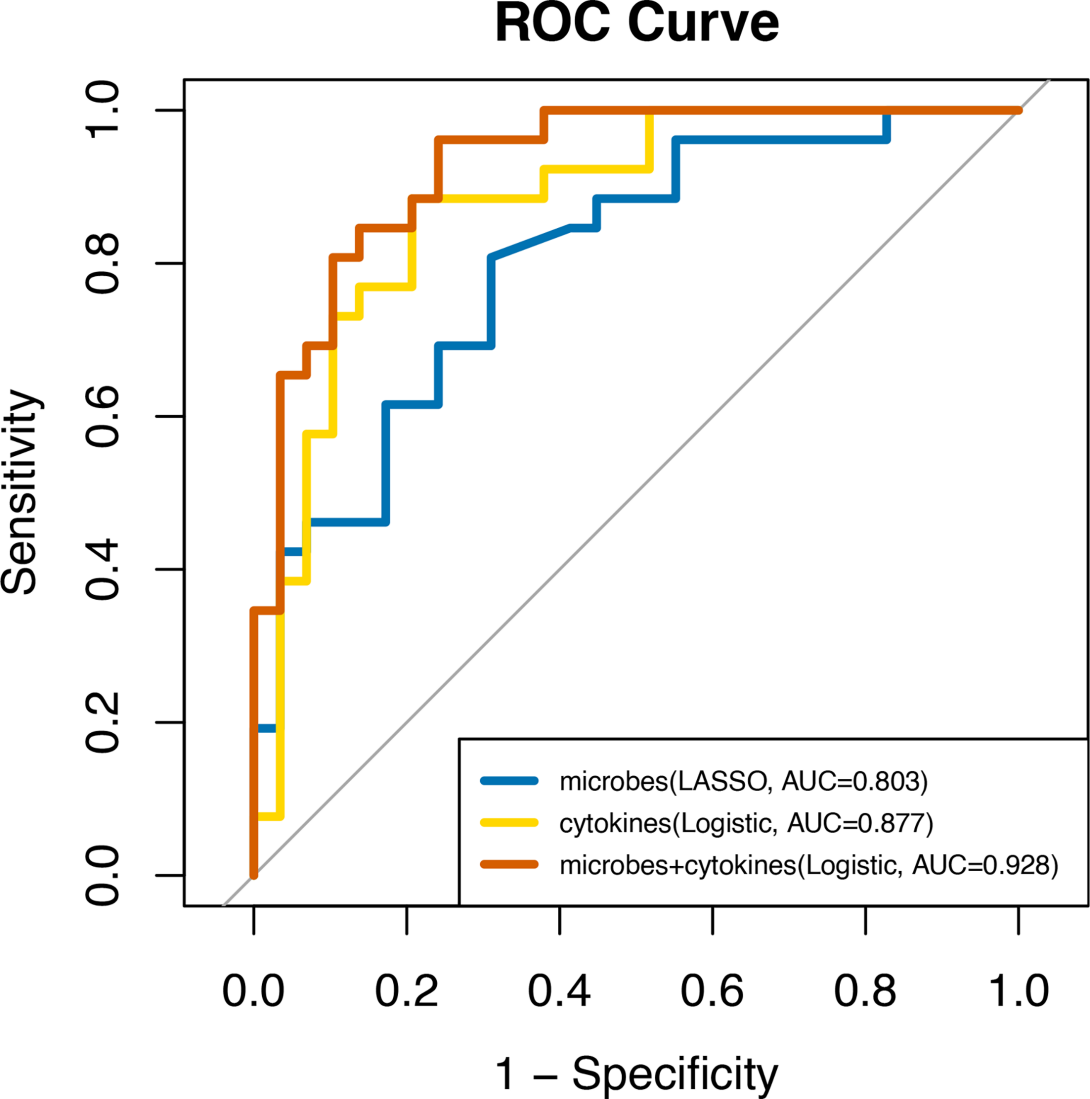
ROC curves for microbes, cytokine and combined models predicting BV recurrence.

Based on these selected features, three predictive models were constructed: a microbes-only model, a cytokine-only model and a combined model integrating both microbial and cytokine features. ROC curve analysis demonstrated that the microbes-based model achieved an AUC of 0.803, while the cytokine-based model yielded an AUC of 0.877. Notably, the combined model exhibited superior discriminative ability, with an AUC of 0.928 (Fig. 5).

To further validate the robustness and generalizability of the combined model, LOOCV was performed. The LOOCV results indicated an AUC of 0.788, an overall accuracy of 0.800, sensitivity of 0.808 and specificity of 0.793, supporting the strong predictive performance of the integrated approach in distinguishing Re-BV cases. Beyond internal statistical validation, we further evaluated the clinical utility of the combined model using a calibration curve and DCA. The calibration curve indicated an acceptable agreement between the predicted probabilities and the actual recurrence outcomes, despite minor fluctuations expected from the limited sample size (Fig. S3a). Importantly, the DCA demonstrated that the combined model provided a positive clinical net benefit across a wide range of threshold probabilities compared to standard baseline strategies (Fig. S3b).

To elucidate the respective predictive contributions of each feature in the combined model, Relative Feature Importance was calculated using Ridge regression (Table S18 and Fig. S4). *Proteus* emerged as the dominant positive driver for predicting recurrence (Relative Importance=100%), followed by *Campylobacter* (18.77%). Notably, the model identified *Howardella* (14.40%) and *Finegoldia* (3.53%) as protective features, negatively contributing to the recurrence probability. Among the cytokines, IL-10 and IL-1β provided minor positive predictive weights, whereas the independent contribution of IL-6 was minimal (<0.01%) when integrated with the dominant microbial signatures.

## Discussion

BV is a common gynaecological disease characterized by an increase in vaginal discharge, which is related to infection, microbial imbalance and oestrogen level [22, 23]. There are a large number of vaginal mucin degrading enzymes in the secretions of BV patients, which disrupt the local mucosal barrier, promote virus adhesion, invasion and even integration into the genome, thereby increasing the risk of HPV infection [24]. The HPV infection rate in BV patients was 21.8% in our cohort, which is consistent with literature reports [25]. Although longitudinal studies have clearly reported an increase in the incidence rate of HPV among BV-positive women, the specific mechanism between HPV infection and BV incidence remains unclear. Our research was limited by the small number of enrolled BV patients and we did not conduct further mechanism exploration.

The characteristic of a healthy vaginal environment is the production of lactic acid by *Lactobacillus*, which plays an important role in eliminating invasive pathogens [8, 13]. Our findings highlight the significant role of *Lactobacillus* as a keystone genus in maintaining a healthy vaginal environment. The gradual decrease in *Lactobacillus* abundance across IBV, Re-BV and BV groups correlates with the progression towards a dysbiotic state. The observed increase in *Gardnerella*, *Prevotella*, *Sneathia*, *Fannyhessea* and *Dialister* in the BV and Re-BV groups supports the hypothesis that these genera may contribute to the pathogenesis of BV. *Gardnerella* is a well-established key pathogen in BV, capable of forming biofilms that protect itself and other anaerobes from host defences and antibiotic treatment. It produces enzymes, such as sialidase and vaginolysin, which can degrade the vaginal epithelial barrier and increase susceptibility to infection [26]. *Prevotella* species are known to produce proteases and lipopolysaccharides that trigger inflammatory responses and synergize with Gardnerella to exacerbate the vaginal dysbiosis [27, 28]. *Sneathia* has been associated with mucosal inflammation, adverse reproductive outcomes and may disrupt the mucosal barrier through activation of immune pathways [29, 30]. *Fannyhessea* (formerly *Atopobium*) contributes to biofilm formation and stability and has been linked to metronidazole resistance, which may play a role in BV recurrence [31, 32]. *Dialister* species may produce short-chain fatty acids and other metabolites that alter the vaginal microenvironment, promoting the growth of other anaerobic bacteria and maintaining the dysbiotic state [33, 34]. The intermediate pattern observed in the IBV group suggests a transitional phase in the microbial community structure, which may reflect the early disturbances that precede the clinical manifestation of BV. These findings highlight the changes in the vaginal microbiota and its response to the development and recurrence of BV.

International guidelines recommend oral or vaginal use of antibiotics (metronidazole, clindamycin or tinidazole) for women diagnosed with BV [35]. The overall clinical effective rate of routine antibiotic treatment for patients with BV is ∼60%, and the disease recurrence rate is between 30 and 40% [36]. 47.3% of BV patients in our study experienced recurrence, which is consistent with the report. The local immune system of the vagina is crucial for maintaining a normal microenvironment. Inflammatory factors play an important role in the formation of vaginosis. A number of studies have reported higher levels of IL-8 in women with BV than in controls with normal Nugent scores [37–39]. Our study showed significantly increased levels of IL-6, IL-1β and IL-10 in BV patients compared to healthy volunteers, in line with prior studies. Ferreira *et al*. found that the levels of IL-6, IL-8 and TNF-α were significantly higher in women with BV than in those with normal Nugent scores [40]. Anahtar *et al*. also reported that the levels of IL-6 and other pro-inflammatory cytokines were elevated in women with BV [41]. These immune signalling molecules originate from antigen-presenting cells, including monocytes and dendritic cells, as well as the vaginal and cervical epithelium [42]. These immune signalling molecules play a crucial role in the immune response to changes in the vaginal microbiome. Some studies have also associated changes in the vaginal microbiome with an increase in the number and activation of T cells, which can worsen inflammation and tissue damage [43, 44].

Notably, the concurrent elevation of pro-inflammatory IL-1β and anti-inflammatory IL-10 in the Re-BV group implies a state of chronic, unresolved inflammation coupled with immune dysregulation. While the persistent dysbiosis drives a continuous inflammatory response (indicated by high IL-1β), the upregulation of IL-10 likely functions as a compensatory negative feedback mechanism. However, this immunosuppressive environment may inadvertently promote recurrence through distinct pathways. Specifically, elevated IL-10 can inhibit the phagocytic activity of macrophages and the Th1-mediated immune response, thereby preventing the complete clearance of residual bacteria [45]. Moreover, such dampened immunity may facilitate the persistence of biofilm-forming pathogens, such as *Gardnerella* spp., allowing them to evade host surveillance and survive antibiotic treatment [46]. This ‘stalemate’ between pathogen persistence and host immune containment might be a hallmark of the recurrent phenotype. In addition, the time to recurrence of BV patients included in this study was within 3 months, so the length of recurrence time may also affect the changes in inflammatory factor levels.

The recurrence of BV remains a major clinical challenge, and currently, there are limited biomarkers available for predicting BV recurrence. Therefore, identifying specific indicators for recurrence prediction is of great clinical significance. Our data demonstrated that a combination of five microbial taxa (*Coriobacteriales bacterium DNF00809*, *Campylobacter*, *Howardella*, *Finegoldia* and *Proteus*) and three cytokines (IL-6, IL-10 and IL-1β) can effectively predict BV recurrence, with an AUC of 0.928 in our cohort. *Coriobacteriales* bacterium *DNF00809* metabolizes formic acid produced by *Gardnerella vaginalis*, helping to regulate vaginal pH and supporting the persistence of BV-associated bacteria. Meanwhile, *Finegoldia magna* contributes to the characteristic fishy odour of BV by producing trimethylamine and may also promote biofilm formation and BV recurrence [47]. Nevertheless, further validation with larger sample sizes is needed to confirm the specificity and sensitivity of these microbial and cytokine markers as predictors for BV recurrence. Furthermore, our feature importance analysis revealed that while specific taxa like *Proteus* strictly drive recurrence, others, such as *Howardella* and *Finegoldia* serve as protective features. This antagonistic dynamic aligns with the complex host-microbe interactions visualized in our correlation heatmap. Although we abstained from embedding mathematical interaction terms into the final prediction equation to prevent severe overfitting given the sample size constraints, the biological synergy between dysbiotic bacteria and pro-inflammatory cytokines clearly underpins the robust performance of our integrated diagnostic approach.

While this study provides novel insights into BV formation and recurrence, certain limitations warrant consideration. First, the sample size was relatively small, particularly for the Re-BV group (*n*=26), and the study lacked an independent external validation cohort as well as longitudinal follow-up data. Consequently, the current model evaluation primarily reflects apparent performance, carrying an inherent risk of over-optimism. Although we strictly utilized LASSO regression combined with LOOCV to mitigate the risk of overfitting and maximize internal predictive stability, the statistical power and clinical generalizability remain constrained. Therefore, the current predictive signature should be interpreted as an exploratory, proof-of-concept framework. Future multi-centre prospective studies with larger independent cohorts and longitudinal designs are essential to externally validate these biomarkers, refine the predictive model and further elucidate the specific immune mechanisms driving BV pathogenesis and recurrence.

Taken together, our findings demonstrate that *Lactobacillus* is the predominant genus responsible for maintaining a healthy vaginal microenvironment. During the progression of BV, the abundance of *Lactobacillus* markedly decreases, accompanied by an increase in diverse anaerobic bacteria, such as *Gardnerella*, *Prevotella*, *Sneathia*, *Fannyhessea* and *Dialister*. Furthermore, the integration of microbiome and cytokine profiles provides a robust predictive model for BV recurrence, highlighting the importance of host-microbe interactions and immune responses in the pathogenesis and recurrence of BV.

## Supplementary material

10.1099/jmm.0.002183 Supplementary Material 1.

## Abbreviations

ASV: amplicon sequence variant
AUC: area under the curve
BV: bacterial vaginosis
DCA: decision curve analysis
HPV: human papillomavirus
IBV: intermediate BV
LDA: linear discriminant analysis
LOOCV: leave-one-out cross-validation
NC: normal control
OTU: operational taxonomic unit
PCoA: principal coordinate analysis
PERMANOVA: permutational multivariate analysis of variance
Re-BV: recurrent BV
ROC: receiver operating characteristic
