# Supplementary material for: Microbiota and inflammatory factor profiles in different stages of bacterial vaginosis formation and recurrence
Source: J Med Microbiol. 2026 Aug 7;75(8):002183. doi: 10.1099/jmm.0.002183 (PMC13450697; doi:10.1099/jmm.0.002183)
Supplement: Supplementary file 1 [file jmm-75-02183-s001.pdf]

# Supplementary Materials

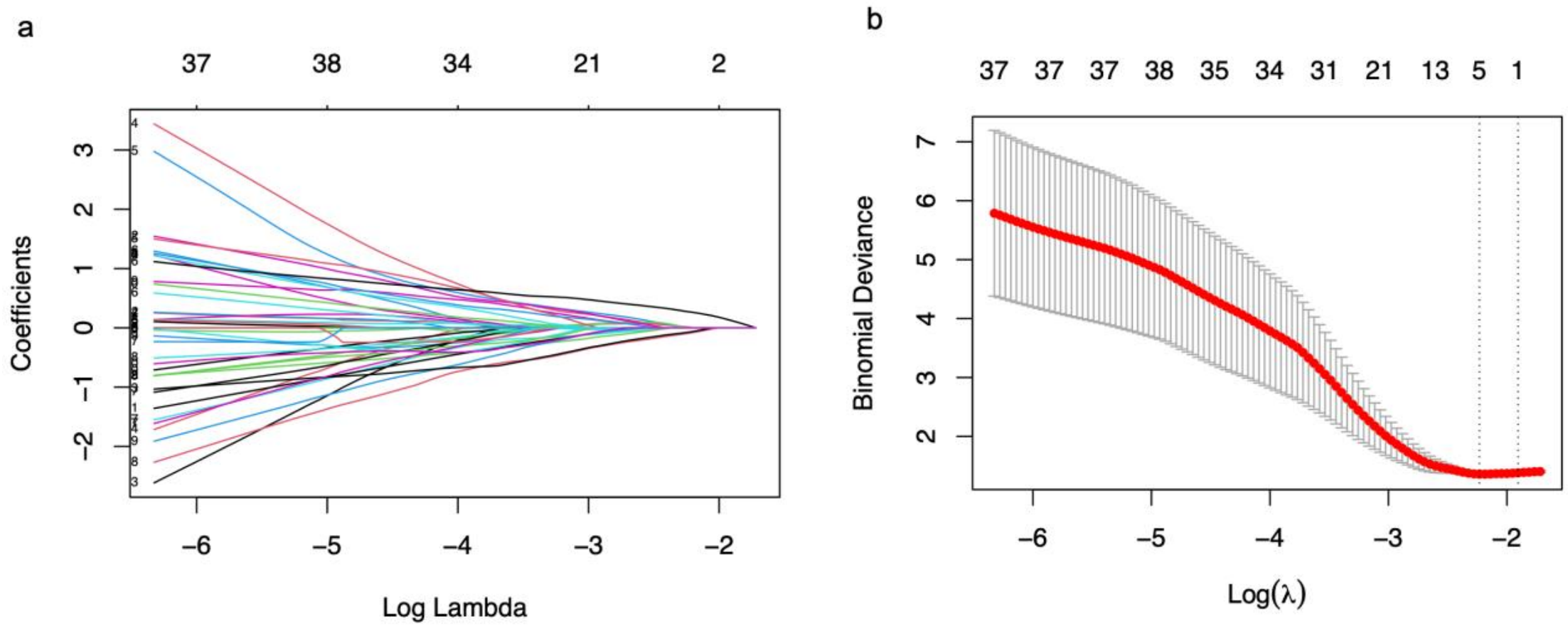


Supplementary Fig S1. LASSO Regression for Microbial Variable Selection (a) Coefficient Paths of Microbial Variables at Different Lambda Values. (b) Cross-Validation Binomial Deviance versus Lambda in LASSO Model.

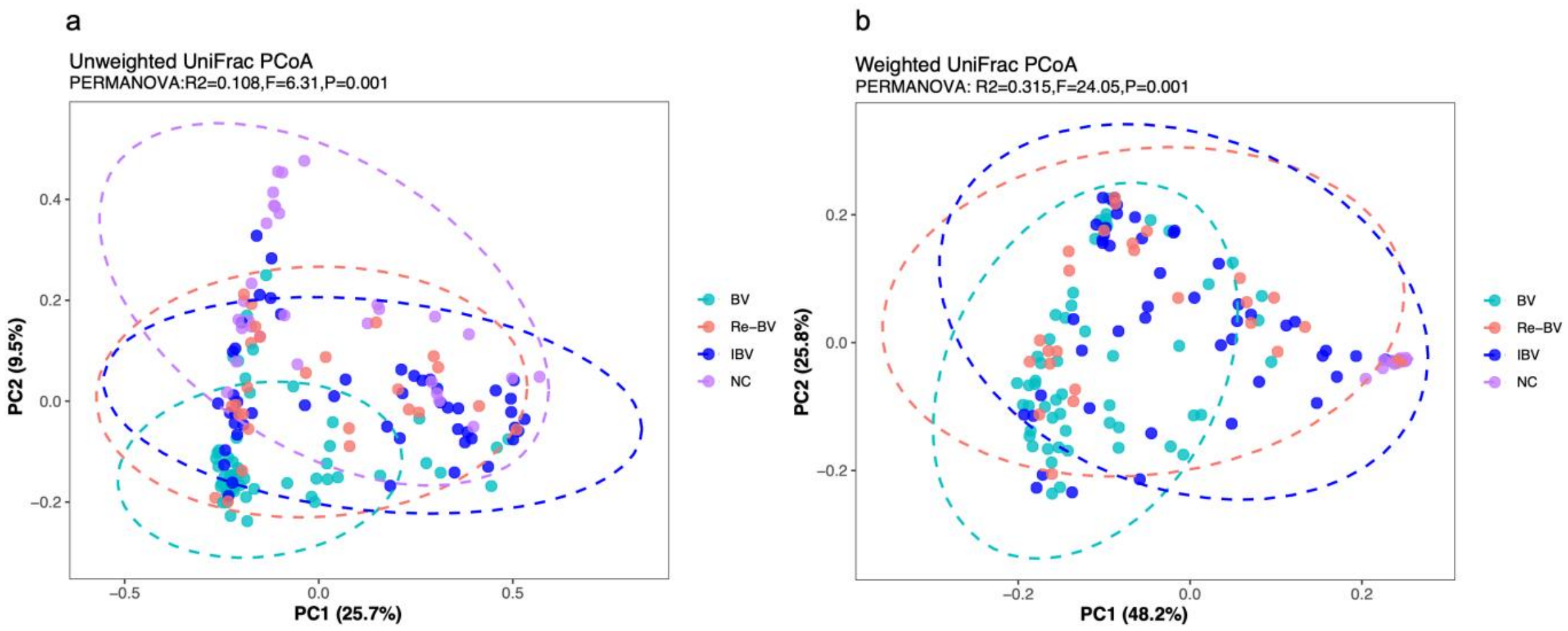


Supplementary Fig S2. Validation of vaginal microbiota beta-diversity using phylogenetic-based distance metrics. (a) PCoA plot based on Unweighted UniFrac distance illustrating the structural variation of microbial communities among the BV, Re-BV, IBV, and NC groups. (b) PCoA plot based on Weighted UniFrac distance further illustrating the community structural variation when accounting for the relative abundance of microbial taxa.

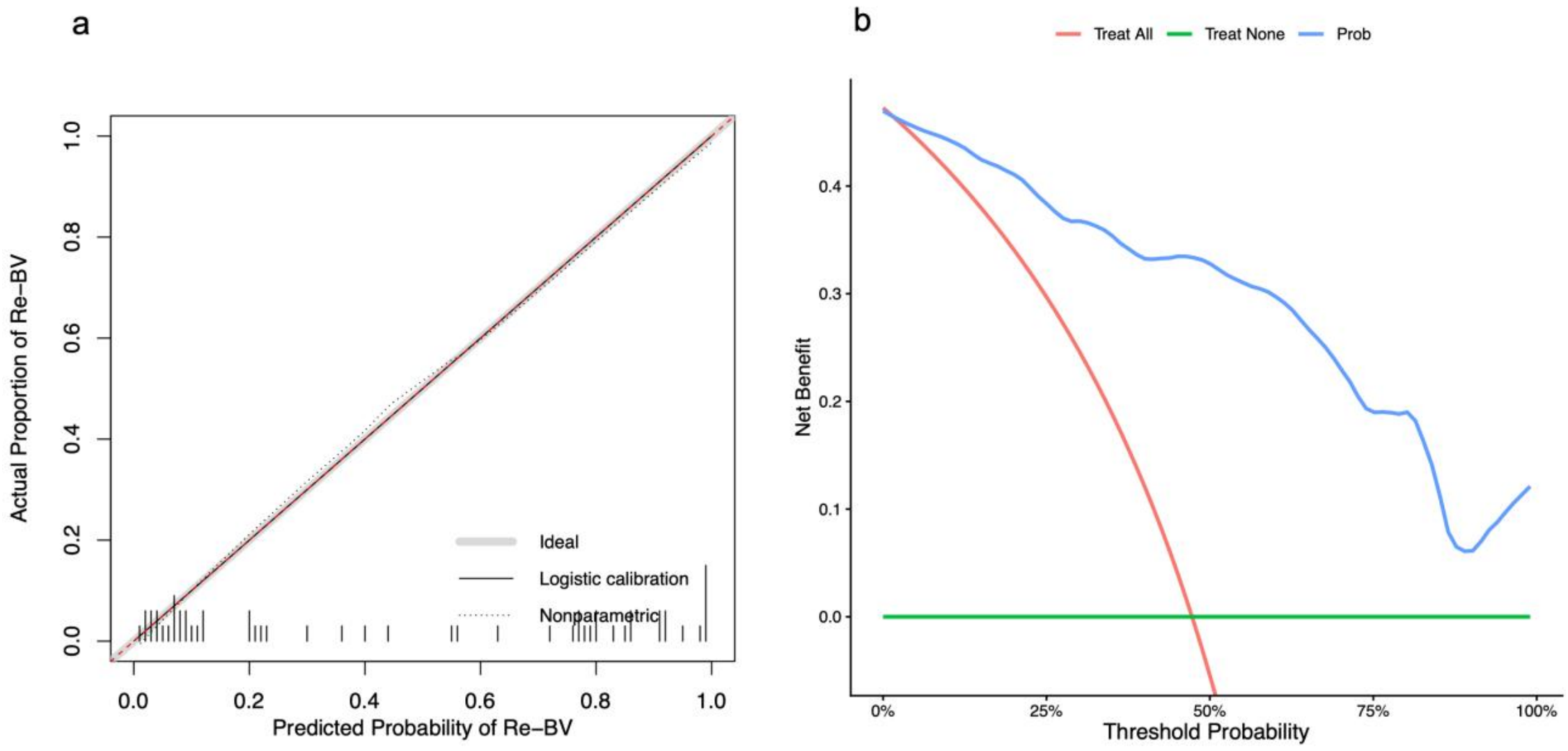


Supplementary Fig S3. Evaluation of the clinical utility and calibration of the joint predictive model. (a) Calibration curve illustrating the agreement between the predicted probabilities of Re-BV and the actual observed proportions. (b) Decision Curve Analysis (DCA) demonstrating that the joint model provides a positive clinical net benefit across a wide range of threshold probabilities.

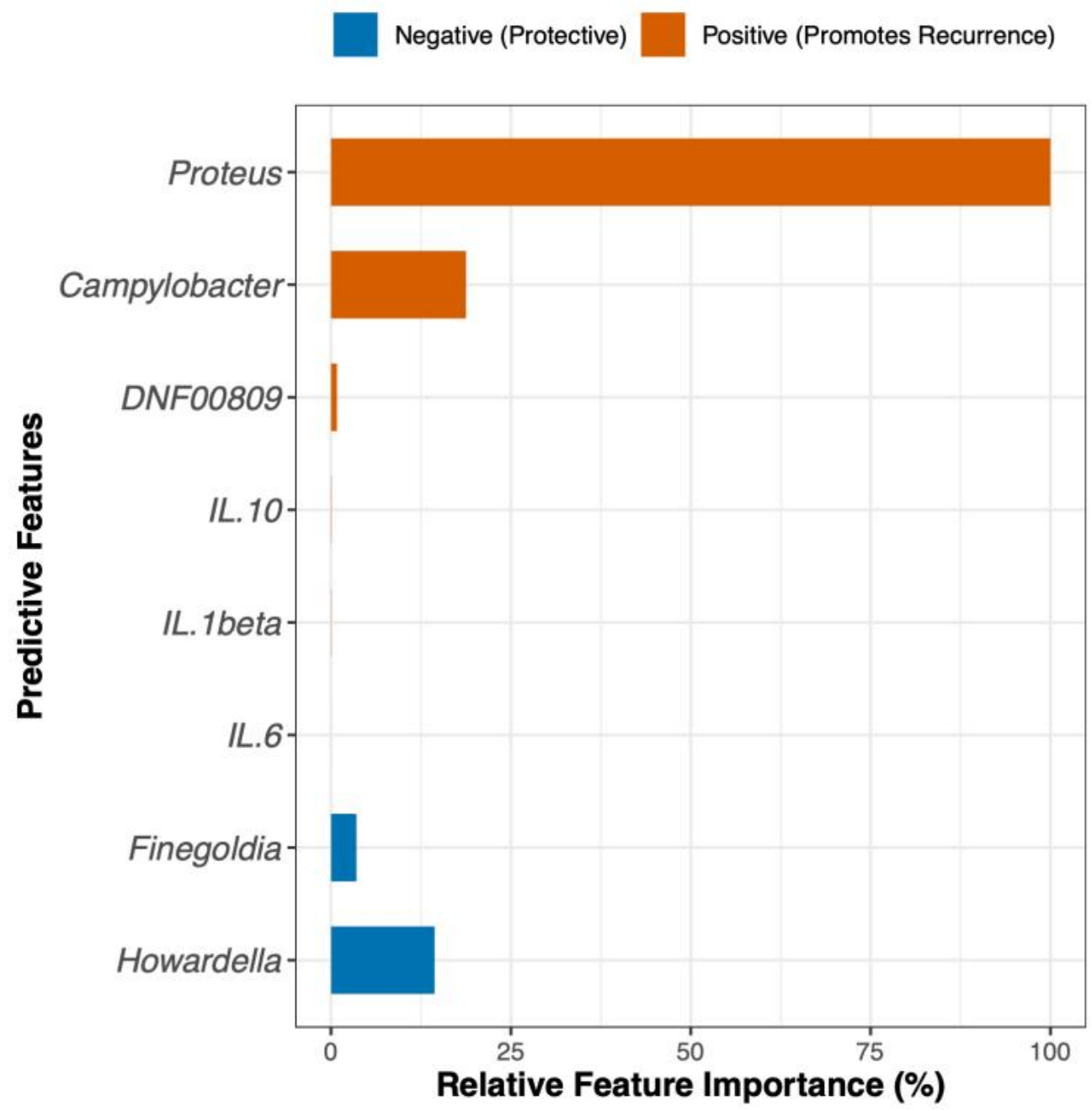


Supplementary Fig S4. Feature importance plot derived from Ridge Regularized Regression. The bar plot illustrates the relative importance of each feature in the combined recurrence model. Red bars indicate positive predictors (risk factors promoting recurrence), while blue bars indicate negative predictors (protective factors against recurrence).

Supplementary Table S1. Pairwise PERMANOVA Analysis of Beta Diversity Among Clinical Groups

| Groups | Df | SumsOfSqs | F.Model | R2 | p.value | p.adjusted |
|---|---|---|---|---|---|---|
| BV vs Re-BV | 1 | 0.68 | 1.84 | 0.02 | 0.019 | 0.0228 |
| BV vs IBV | 1 | 1.32 | 3.51 | 0.03 | 0.001 | 0.0015 |
| BV vs NC | 1 | 8.54 | 29.69 | 0.26 | 0.001 | 0.0015 |
| Re-BV vs IBV | 1 | 0.53 | 1.35 | 0.02 | 0.154 | 0.1540 |
| Re-BV vs NC | 1 | 5.11 | 19.58 | 0.27 | 0.001 | 0.0015 |
| IBV vs NC | 1 | 7.03 | 23.36 | 0.23 | 0.001 | 0.0015 |

Note: Pairwise differences in microbial community structure among clinical groups were assessed using PERMANOVA (Adonis) based on Bray-Curtis distances. P-values were adjusted for multiple comparisons using the Benjamini-Hochberg method.

Supplementary Table S2. Pairwise PERMANOVA Analysis of Beta Diversity Based on Unweighted UniFrac Distances Among Clinical Groups

| Groups | Sample size | Permutations | pseudo-F | p-value | q-value |
|---|---|---|---|---|---|
| BV vs Re-BV | 81 | 999 | 5.356 | 0.001 | 0.0012 |
| BV vs IBV | 105 | 999 | 8.457 | 0.001 | 0.0012 |
| BV vs NC | 85 | 999 | 10.669 | 0.001 | 0.0012 |
| Re-BV vs IBV | 76 | 999 | 3.258 | 0.003 | 0.003 |
| Re-BV vs NC | 56 | 999 | 4.118 | 0.001 | 0.0012 |
| IBV vs NC | 80 | 999 | 4.613 | 0.001 | 0.0012 |

Note: Pairwise differences in microbial community structure among clinical groups were assessed using PERMANOVA (Adonis) based on Unweighted UniFrac distances. P-values were adjusted for multiple comparisons using the Benjamini-Hochberg method to generate q-values.

Supplementary Table S3. Pairwise PERMANOVA Analysis of Beta Diversity Based on Weighted UniFrac Distances Among Clinical Groups

| Groups | Sample size | Permutations | pseudo-F | p-value | q-value |
|---|---|---|---|---|---|
| BV vs Re-BV | 81 | 999 | 4.202 | 0.004 | 0.0048 |
| BV vs IBV | 105 | 999 | 7.267 | 0.001 | 0.0015 |
| BV vs NC | 85 | 999 | 82.859 | 0.001 | 0.0015 |
| Re-BV vs IBV | 76 | 999 | 1.081 | 0.363 | 0.363 |
| Re-BV vs NC | 56 | 999 | 64.127 | 0.001 | 0.0015 |
| IBV vs NC | 80 | 999 | 40.091 | 0.001 | 0.0015 |

Note: Pairwise differences in microbial community structure among clinical groups were assessed using PERMANOVA (Adonis) based on Weighted UniFrac distances. P-values were adjusted for multiple comparisons using the Benjamini-Hochberg method to generate q-values.

Supplementary Table S4. Spearman correlation analysis between vaginal bacterial genera abundance and vaginal pH

| Genus | Spearman's rho | p-value |
|---|---|---|
| *Lactobacillus* | -0.39 | 2.816E-07 |
| *Gardnerella* | 0.24 | 2.578E-03 |
| *Prevotella* | 0.37 | 1.746E-06 |
| *Sneathia* | 0.29 | 1.584E-04 |
| *Fannyhessea* | 0.32 | 4.017E-05 |
| *Dialister* | 0.35 | 4.637E-06 |

Supplementary Table S5. Spearman correlation between genus abundance and age

| Genus | Spearman's rho | p-value |
|---|---|---|
| *Lactobacillus* | -0.0506 | 0.5242 |
| *Gardnerella* | 0.0540 | 0.4960 |
| *Prevotella* | -0.0005 | 0.9950 |
| *Sneathia* | 0.0154 | 0.8459 |
| *Fannyhessea* | 0.0080 | 0.9199 |
| *Dialister* | -0.0266 | 0.7373 |

Supplementary Table S6. Comparison of genus abundance between HPV-negative and HPV-positive groups

| Genus | HPV-negative Median (%) | HPV-positive Median (%) | Mann-Whitney U Statistic (W) | p-value |
|---|---|---|---|---|
| *Lactobacillus* | 15.12 | 1.30 | 1617 | 0.0099 |
| *Gardnerella* | 21.18 | 12.77 | 1253 | 0.6011 |
| *Prevotella* | 0.52 | 11.84 | 581 | 0.0010 |
| *Sneathia* | 0.14 | 4.07 | 926 | 0.1748 |
| *Fannyhessea* | 0.72 | 6.50 | 748 | 0.0196 |
| *Dialister* | 0.33 | 1.63 | 804 | 0.0416 |

Supplementary Table S7. Comparison of genus abundance by hydrogen peroxide status

| Genus | hydrogen peroxide -negative Median (%) | hydrogen peroxide -positive Median (%) | Mann-Whitney U Statistic (W) | p-value |
|---|---|---|---|---|
| *Lactobacillus* | 63.51 | 7.39 | 1571 | 0.1283 |
| *Gardnerella* | 4.13 | 24.39 | 598 | 0.0002 |
| *Prevotella* | 0.10 | 0.75 | 1074 | 0.2523 |
| *Sneathia* | 1.45 | 0.14 | 1580 | 0.1066 |
| *Fannyhessea* | 0.63 | 1.03 | 1201 | 0.6453 |
| *Dialister* | 0.32 | 0.55 | 1221 | 0.7215 |

Supplementary Table S8. Comparison of genus abundance by leukocyte esterase status

| Genus | leukocyte esterase-negative Median (%) | leukocyte esterase-positive Median (%) | Mann-Whitney U Statistic (W) | p-value |
|---|---|---|---|---|
| *Lactobacillus* | 16.28 | 3.12 | 2479 | 0.0795 |
| *Gardnerella* | 18.11 | 26.82 | 1719 | 0.1440 |
| *Prevotella* | 0.40 | 7.85 | 1286 | 0.0009 |
| *Sneathia* | 0.14 | 0.22 | 1692 | 0.1060 |
| *Fannyhessea* | 0.74 | 3.56 | 1797 | 0.2570 |
| *Dialister* | 0.22 | 2.34 | 1136 | 0.0001 |

Supplementary Table S9. Comparison of genus abundance by neuraminidase status

| Genus | neuraminidase-negative Median (%) | neuraminidase-positive Median (%) | Mann-Whitney U Statistic (W) | p-value |
|---|---|---|---|---|
| *Lactobacillus* | 41.70 | 2.87 | 4214 | 1.580E-05 |
| *Gardnerella* | 12.73 | 23.88 | 2391 | 3.600E-02 |
| *Prevotella* | 0.33 | 8.70 | 1423 | 3.210E-08 |
| *Sneathia* | 0.09 | 2.62 | 1972 | 2.390E-04 |
| *Fannyhessea* | 0.53 | 6.78 | 1694 | 4.930E-06 |
| *Dialister* | 0.05 | 2.06 | 1259 | 6.800E-10 |

Supplementary Table S10. Comparison of genus abundance by Acetylglucosaminidase status

| Genus | Acetylglucosaminidase-negative Median (%) | Acetylglucosaminidase-positive Median (%) | Mann-Whitney U Statistic (W) | p-value |
|---|---|---|---|---|
| *Lactobacillus* | 35.96 | 3.04 | 3809 | 2.833E-03 |
| *Gardnerella* | 18.21 | 21.18 | 2589 | 1.849E-01 |
| *Prevotella* | 0.38 | 4.06 | 1971 | 4.257E-04 |
| *Sneathia* | 0.07 | 0.43 | 2065 | 1.090E-03 |
| *Fannyhessea* | 0.59 | 5.82 | 1945 | 3.024E-04 |
| *Dialister* | 0.14 | 1.85 | 1741 | 1.167E-05 |

Note: All statistical analyses in Supplementary Table S2-S8 were performed using appropriate non-parametric tests (Spearman correlation or Mann-Whitney U test, as indicated).

Supplementary Table S11. Estimated marginal means and 95% confidence intervals of IL-6 levels in each group

| Group | Emmean | SE | Lower.CL | Upper.CL |
|---|---|---|---|---|
| BV | 180 | 8.15 | 164 | 196 |
| IBV | 170 | 9.88 | 151 | 190 |
| NC | 129 | 11.5 | 107 | 152 |

| | | | | |
|---|---|---|---|---|
| Re-BV | 165 | 12.5 | 140 | 189 |

Supplementary Table S12. Pairwise comparisons of IL-6 levels among groups (Tukey-adjusted)

| Contrast | Estimate | SE | t.ratio | p.value |
|---|---|---|---|---|
| BV vs IBV | 9.7 | 10.1 | 0.962 | 0.771 |
| BV vs NC | 50.74 | 12.5 | 4.051 | 0.0005 |
| BV vs Re-BV | 15.47 | 12.4 | 1.251 | 0.595 |
| IBV vs NC | 41.04 | 12.8 | 3.207 | 0.0088 |
| IBV vs Re-BV | 5.77 | 12.2 | 0.474 | 0.965 |
| NC vs Re-BV | -35.27 | 14.5 | -2.428 | 0.076 |

Supplementary Table S13. Estimated marginal means and 95% confidence intervals of IL-10 levels in each group

| Group | Emmean | SE | Lower.CL | Upper.CL |
|---|---|---|---|---|
| BV | 171 | 12.2 | 146.9 | 194.9 |
| IBV | 161 | 14.7 | 132 | 190.2 |
| NC | 66 | 17.1 | 32.3 | 99.8 |
| Re-BV | 271 | 18.6 | 234.5 | 308.2 |

Supplementary Table S14. Pairwise comparisons of IL-10 levels among groups (Tukey-adjusted)

| Contrast | Estimate | SE | t.ratio | p.value |
|---|---|---|---|---|
| BV vs IBV | 9.8 | 15 | 0.652 | 0.915 |
| BV vs NC | 104.8 | 18.7 | 5.615 | <0.0001 |
| BV vs Re-BV | -100.5 | 18.4 | -5.451 | <0.0001 |
| IBV vs NC | 95 | 19.1 | 4.982 | <0.0001 |
| IBV vs Re-BV | -110.3 | 18.1 | -6.076 | <0.0001 |
| NC vs Re-BV | -205.3 | 21.7 | -9.48 | <0.0001 |

Supplementary Table S15. Estimated marginal means and 95% confidence intervals of IL-1β levels in each group

| Group | Emmean | SE | Lower.CL | Upper.CL |
|---|---|---|---|---|
| BV | 123.8 | 5.56 | 112.8 | 135 |
| IBV | 116.1 | 6.74 | 102.8 | 129 |
| NC | 86.1 | 7.82 | 70.7 | 102 |
| Re-BV | 193.2 | 8.53 | 176.4 | 210 |

Supplementary Table S16. Pairwise comparisons of IL-1β levels among groups (Tukey-adjusted)

| Contrast | Estimate | SE | t.ratio | p.value |
|---|---|---|---|---|
| BV vs IBV | 7.67 | 6.88 | 1.115 | 0.681 |

| | | | | |
|---|---|---|---|---|
| BV vs NC | 37.66 | 8.55 | 4.406 | 0.0001 |
| BV vs Re-BV | -69.46 | 8.44 | -8.232 | <0.0001 |
| IBV vs NC | 29.99 | 8.73 | 3.435 | 0.0042 |
| IBV vs Re-BV | -77.12 | 8.31 | -9.284 | <0.0001 |
| NC vs Re-BV | -107.12 | 9.91 | -10.805 | <0.0001 |

Supplementary Table S17. Microbial Features Selected by LASSO Regression and Their Coefficients in Predicting BV Recurrence

| feature | coef |
|---|---|
| *DNF00809* | 0.0049 |
| *Campylobacter* | 0.0132 |
| *Howardella* | -0.0907 |
| *Finegoldia* | -0.0730 |
| *Proteus* | 0.2705 |

Note: "*DNF00809*" refers to *Coriobacteriales* bacterium *DNF00809*.

Supplementary Table S18. Relative feature importance of the selected microbes and inflammatory factors in the combined predictive model, derived from Ridge Regularized Regression.

| Predictive Feature | Relative Importance (%) | Clinical Effect |
|---|---|---|
| *Proteus* | 100 | Promotes Recurrence |
| *Campylobacter* | 18.77 | Promotes Recurrence |
| *Howardella* | 14.4 | Protective |
| *Finegoldia* | 3.53 | Protective |
| *DNF00809* | 0.82 | Promotes Recurrence |
| IL.10 | 0.05 | Promotes Recurrence |
| IL.1beta | 0.05 | Promotes Recurrence |
| IL.6 | <0.01 | Promotes Recurrence |

Supplementary Table S19. Experiment Accession Numbers

| Rank | Sample ID | Accession |
|---|---|---|
| 1 | BV1 | SRX26088591 |
| 2 | BV2 | SRX26088592 |
| 3 | BV3 | SRX26088664 |
| 4 | BV4 | SRX26088675 |
| 5 | BV5 | SRX26088686 |
| 6 | BV6 | SRX26088697 |
| 7 | BV7 | SRX26088708 |
| 8 | BV8 | SRX26088719 |
| 9 | BV9 | SRX26088730 |
| 10 | BV10 | SRX26088741 |
| 11 | BV11 | SRX26088593 |
| 12 | BV12 | SRX26088604 |
| 13 | BV13 | SRX26088615 |
| 14 | BV14 | SRX26088626 |
| 15 | BV15 | SRX26088637 |
| 16 | BV16 | SRX26088648 |
| 17 | BV17 | SRX26088659 |
| 18 | BV18 | SRX26088661 |
| 19 | BV19 | SRX26088662 |
| 20 | BV20 | SRX26088663 |
| 21 | BV21 | SRX26088665 |
| 22 | BV22 | SRX26088666 |
| 23 | BV23 | SRX26088667 |
| 24 | BV24 | SRX26088668 |
| 25 | BV25 | SRX26088669 |
| 26 | BV26 | SRX26088670 |
| 27 | BV27 | SRX26088671 |
| 28 | BV28 | SRX26088672 |
| 29 | BV29 | SRX26088673 |
| 30 | BV30 | SRX26088674 |
| 31 | BV31 | SRX26088676 |
| 32 | BV32 | SRX26088677 |
| 33 | BV33 | SRX26088678 |
| 34 | BV34 | SRX26088679 |
| 35 | BV35 | SRX26088680 |
| 36 | BV36 | SRX26088681 |
| 37 | BV37 | SRX26088682 |

| | | |
|---|---|---|
| 38 | BV38 | SRX26088683 |
| 39 | BV39 | SRX26088684 |
| 40 | BV40 | SRX26088685 |
| 41 | BV41 | SRX26088687 |
| 42 | BV42 | SRX26088688 |
| 43 | BV43 | SRX26088689 |
| 44 | BV44 | SRX26088690 |
| 45 | BV45 | SRX26088691 |
| 46 | BV46 | SRX26088692 |
| 47 | BV47 | SRX26088693 |
| 48 | BV48 | SRX26088694 |
| 49 | BV49 | SRX26088695 |
| 50 | BV50 | SRX26088696 |
| 51 | BV51 | SRX26088698 |
| 52 | BV52 | SRX26088699 |
| 53 | BV53 | SRX26088700 |
| 54 | BV54 | SRX26088701 |
| 55 | BV55 | SRX26088702 |
| 56 | Re-BV1 | SRX26088703 |
| 57 | Re-BV2 | SRX26088704 |
| 58 | Re-BV3 | SRX26088705 |
| 59 | Re-BV4 | SRX26088706 |
| 60 | Re-BV5 | SRX26088707 |
| 61 | Re-BV6 | SRX26088709 |
| 62 | Re-BV7 | SRX26088710 |
| 63 | Re-BV8 | SRX26088711 |
| 64 | Re-BV9 | SRX26088712 |
| 65 | Re-BV10 | SRX26088713 |
| 66 | Re-BV11 | SRX26088714 |
| 67 | Re-BV12 | SRX26088715 |
| 68 | Re-BV13 | SRX26088716 |
| 69 | Re-BV14 | SRX26088717 |
| 70 | Re-BV15 | SRX26088718 |
| 71 | Re-BV16 | SRX26088720 |
| 72 | Re-BV17 | SRX26088721 |
| 73 | Re-BV18 | SRX26088722 |
| 74 | Re-BV19 | SRX26088723 |
| 75 | Re-BV20 | SRX26088724 |
| 76 | Re-BV21 | SRX26088725 |

| | | |
|---|---|---|
| 77 | Re-BV22 | SRX26088726 |
| 78 | Re-BV23 | SRX26088727 |
| 79 | Re-BV24 | SRX26088728 |
| 80 | Re-BV25 | SRX26088729 |
| 81 | Re-BV26 | SRX26088731 |
| 82 | IBV1 | SRX26088732 |
| 83 | IBV2 | SRX26088733 |
| 84 | IBV3 | SRX26088734 |
| 85 | IBV4 | SRX26088735 |
| 86 | IBV5 | SRX26088736 |
| 87 | IBV6 | SRX26088737 |
| 88 | IBV7 | SRX26088738 |
| 89 | IBV8 | SRX26088739 |
| 90 | IBV9 | SRX26088740 |
| 91 | IBV10 | SRX26088742 |
| 92 | IBV11 | SRX26088743 |
| 93 | IBV12 | SRX26088744 |
| 94 | IBV13 | SRX26088745 |
| 95 | IBV14 | SRX26088746 |
| 96 | IBV15 | SRX26088747 |
| 97 | IBV16 | SRX26088748 |
| 98 | IBV17 | SRX26088749 |
| 99 | IBV18 | SRX26088750 |
| 100 | IBV19 | SRX26088751 |
| 101 | IBV20 | SRX26088594 |
| 102 | IBV21 | SRX26088595 |
| 103 | IBV22 | SRX26088596 |
| 104 | IBV23 | SRX26088597 |
| 105 | IBV24 | SRX26088598 |
| 106 | IBV25 | SRX26088599 |
| 107 | IBV26 | SRX26088600 |
| 108 | IBV27 | SRX26088601 |
| 109 | IBV28 | SRX26088602 |
| 110 | IBV29 | SRX26088603 |
| 111 | IBV30 | SRX26088605 |
| 112 | IBV31 | SRX26088606 |
| 113 | IBV32 | SRX26088607 |
| 114 | IBV33 | SRX26088608 |
| 115 | IBV34 | SRX26088609 |

| | | |
|---|---|---|
| 116 | IBV35 | SRX26088610 |
| 117 | IBV36 | SRX26088611 |
| 118 | IBV37 | SRX26088612 |
| 119 | IBV38 | SRX26088613 |
| 120 | IBV39 | SRX26088614 |
| 121 | IBV40 | SRX26088616 |
| 122 | IBV41 | SRX26088617 |
| 123 | IBV42 | SRX26088618 |
| 124 | IBV43 | SRX26088619 |
| 125 | IBV44 | SRX26088620 |
| 126 | IBV45 | SRX26088621 |
| 127 | IBV46 | SRX26088622 |
| 128 | IBV47 | SRX26088623 |
| 129 | IBV48 | SRX26088624 |
| 130 | IBV49 | SRX26088625 |
| 131 | IBV50 | SRX26088627 |
| 132 | NC1 | SRX26088628 |
| 133 | NC2 | SRX26088629 |
| 134 | NC3 | SRX26088630 |
| 135 | NC4 | SRX26088631 |
| 136 | NC5 | SRX26088632 |
| 137 | NC6 | SRX26088633 |
| 138 | NC7 | SRX26088634 |
| 139 | NC8 | SRX26088635 |
| 140 | NC9 | SRX26088636 |
| 141 | NC10 | SRX26088638 |
| 142 | NC11 | SRX26088639 |
| 143 | NC12 | SRX26088640 |
| 144 | NC13 | SRX26088641 |
| 145 | NC14 | SRX26088642 |
| 146 | NC15 | SRX26088643 |
| 147 | NC16 | SRX26088644 |
| 148 | NC17 | SRX26088645 |
| 149 | NC18 | SRX26088646 |
| 150 | NC19 | SRX26088647 |
| 151 | NC20 | SRX26088649 |
| 152 | NC21 | SRX26088650 |
| 153 | NC22 | SRX26088651 |
| 154 | NC23 | SRX26088652 |

| | | |
|---|---|---|
| 155 | NC24 | SRX26088653 |
| 156 | NC25 | SRX26088654 |
| 157 | NC26 | SRX26088655 |
| 158 | NC27 | SRX26088656 |
| 159 | NC28 | SRX26088657 |
| 160 | NC29 | SRX26088658 |
| 161 | NC30 | SRX26088660 |
